# Role of autophagy in atherosclerosis: foe or friend?

**DOI:** 10.1186/s12950-019-0212-4

**Published:** 2019-05-02

**Authors:** Mehdi Hassanpour, Reza Rahbarghazi, Mohammad Nouri, Nasser Aghamohammadzadeh, Nasser Safaei, Mahdi Ahmadi

**Affiliations:** 1 Stem Cell And Regenerative Medicine Institute, Tabriz University of Medical Sciences, Tabriz, Iran; 2 Stem Cell Research Center, Tabriz University of Medical Sciences, Tabriz, Iran; 3 Department of Applied Cell Sciences, Faculty of Advanced Medical Sciences, Tabriz University of Medical Sciences, Tabriz, Iran; 4 Endocrine Research Center, Tabriz University of Medical Sciences, Tabriz, Iran; 5 Cardiovascular Research Center, Tabriz University of Medical Sciences, Tabriz, Iran; 6 Department of Cardiac Surgery, Shahid Madani Heart Hospital, Tabriz University of Medical Sciences, Tabriz, Iran; 7 Department of Physiology, Faculty of Medicine, Tabriz University of Medical Sciences, Tabriz, Iran

**Keywords:** Autophagy, AS, Pharmacological intervention

## Abstract

Athrosclerosis is conceived as a chronic inflammatory status affecting cells from vascular walls. Different mechanisms and pathological features are evident at the onset of atherosclerotic changes via the engaging different cells from the vascular wall and circulatory cells. Attempts are currently focused on the detection of cell compensatory mechanisms against atherosclerotic changes to restore cell function and/or postpone severe vasculitis. Autophagy is an intracellular self-digesting process commonly protrudes exhausted organelles and injured cytoplasmic constituents via double-lipid bilayer membrane vesicles out the target cells. Recent investigations point to the critical and defensive role of autophagy in the vascular cells behavioral function such as endothelial cells and smooth muscle cells against different insults. Autophagy response and related effectors could be modulated in the favor to restore cell function and reduce pro-inflammatory status under pathological conditions. In this review, the recent findings were collected regarding the role of autophagy during atherosclerotic changes. We aimed to answer the question of how autophagy stimulation and/or inhibition could provide a promising effect on developing a sophisticated treatment for AS.

## Introduction

AS is touted as a chronic inflammatory disease affecting cells from vascular walls. These changes lead to the emergence of atherosclerotic plaques in the luminal surface and conceived as a major cause of CVD. CVDs are responsible for 31% of all global deaths [1]. Formation of atherosclerotic plaques is classified into four distinct stages: fatty streak, atheromatous plaque, complicated atheromatous plaque and clinical complications in clinical pathology. The complicated atheromatous plaque has been referred to unstable plaques which are characterized by the accumulation of foam cells in a thin fibrous cap. Uncontrolled destruction or detachments of plaques are the main cause of acute cardiovascular events. It has been reported that a series of events participate to accelerate plaque rupture. The exact mechanisms involved in plaque rupture are unknown but some condition such as lipid deposition, oxidative stress, inflammation, endothelial dysfunction, foam cell formation, and SMC differentiation could be exemplified. The plaques are detached by macrophages via releasing various pro-inflammatory factors and matrix catabolic enzymes; resulting in SMC death and plaques instability [2]. The exposure of collagen fiber to degrading enzymes further loosens the trap plaques. The induction of pro-inflammatory factors such as IL-1, − 6, − 12 along with TNF-α promotes additional inflammatory responses in atherosclerotic plaques [3].

### Biology of autophagy

**Fig. 1:**
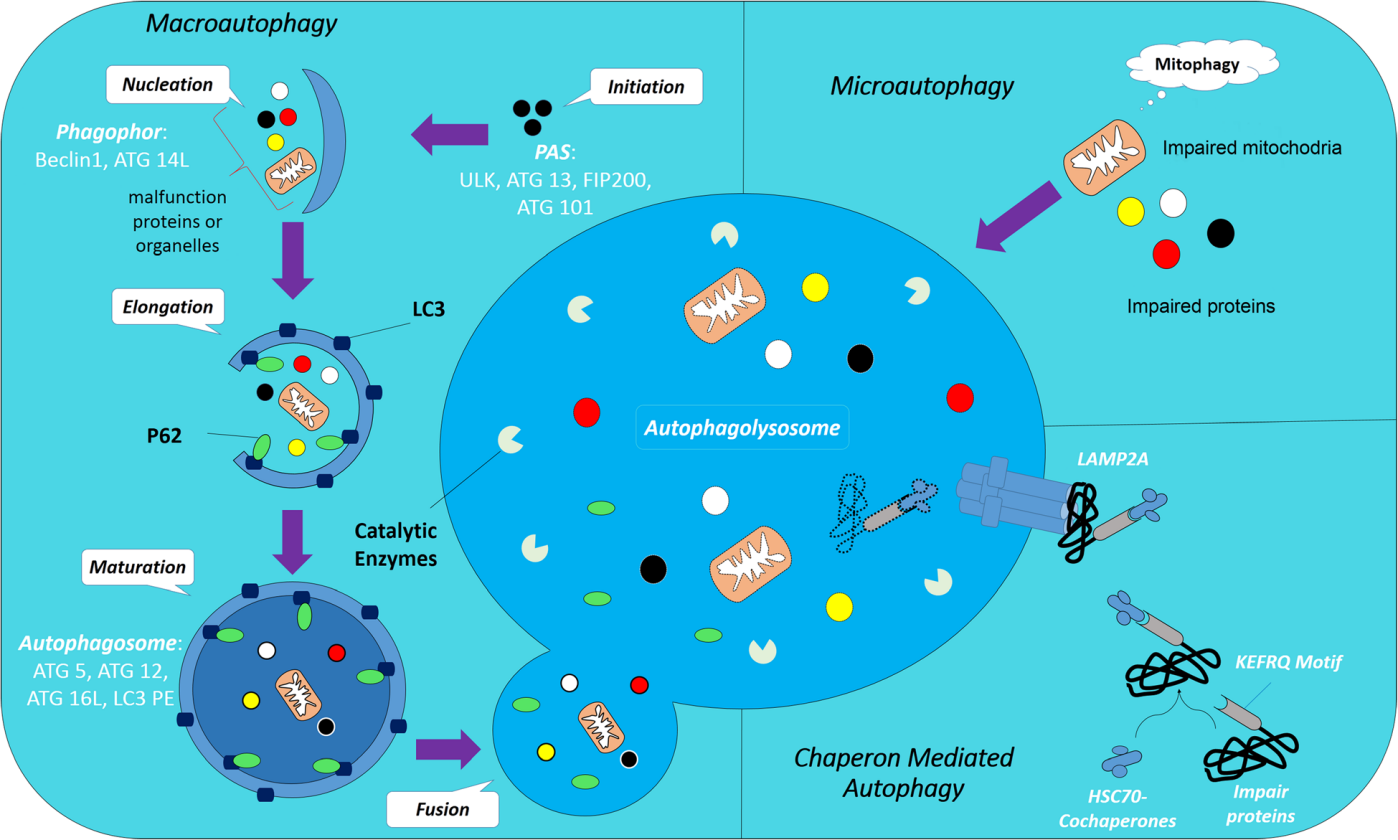
Autophagy process involves three distinct types by route of delivery: Macroautophagy, microautophagy, and chaperone-mediated autophagy (CMA). Autophagy is started by the nucleation and isolation of membrane named phagophore. The isolated membrane is then elongated and engulfed on itself to form an autophagosome. Autophagosomes then fuse with lysosomes and forming the autophagolysosomes. The conversion of LC3-I to LC3-Il caused to the formation of autophagolysosomes and P62 facilitates the docking of cargo to the cell membrane during the autophagic response. In microautophagy, substances directly enter into the lysosomes through the intrusion of self-membrane and finally contributed to autophagy-related pathways

The kinetics of protein (degradation and/or synthesis) is significant to the physiological activity of all cell types. It is believed that the ubiquitin-proteasome system along with autophagy is two major strategies to destroy intracellular damaged proteins. Autophagy is a sophisticated complex intracellular process that carries and degrades impaired proteins or whole dysfunction organelles by fusion to lysosomes and action of hydrolytic enzymes [4, 5]. Based on the route of delivery, there are three types of autophagy exist inside of cells follows as; I) Macroautophagy: the exhausted materials are enclosed with a dual lipid membrane namely autophagosomes and then fuse with lysosomes to form autophagolysosome II) Microautophagy directly enters substances into the lysosomes through the intrusion of self-membrane and finally III) CMA degrades target molecules by engaging specific motifs (KFERQ) targeted by HSC70 complex and then adhere to lysosome via LAMP2A. Based on molecules, autophagy is divided into aggrephagy, mitophagy, lipophagy, and xenophagy depending on which substance is sequestrated and digested [6]. In this review, macroautophagy will be explained as autophagy (Fig. 1).

In most tissues, autophagy occurs at a basic level for sustained metabolic recycling of intracellular components, but during the occurrence of adverse situations such as stress-related signals autophagy is activated and required for cells survival and recycling nutrients [7]. Since autophagy is involved in the prevention of various pathological diseases, detection of autophagic underlying mechanisms is very critical. The lack of Atg in the whole body or specific tissues in can cause serious disability and death of organisms and this strongly supports the hypothesis that autophagy is an important step in maintaining health [8]. Although a number of studies have been done to demonstrate that autophagy protects a variety of physiological processes and diseases, the role of autophagy in CVD is not well known yet [9].

AS is a chronic inflammatory disease of the arterial wall and is known to be one of the major risk factor leading to CVDs [10]. Thus, it is necessary to understand the basic process of plaque formation and rupture in order to develop better treatment methods. Recent evidence has demonstrated that autophagy plays an important role in regulating AS and possibly will provide new opportunities for the treatment of an inflammatory condition [11, 12].

### The role of autophagy in the promotion/reduction of inflammation

With regard to the chronic status of atherosclerotic changes, an exaggerated inflammatory activation may cause to local proteolysis, plaque instability, and rupture, and develop of thrombus, which causes ischemic heart disease and MI. Already, numerous inflammatory markers were targeted to monitoring of progression of AS and anti-inflammatory therapy may have efficacy to control pathological changes in the vasculature wall. An increasing number of investigations have confirmed that autophagy has significant effects on the stimulation of the inflammatory responses [13]. Nevertheless, when the inflammatory reaction is not appropriately controlled, autophagy can be detrimental for the host. A reciprocal relationship has been already identified between autophagic status and inflammation. Autophagy, by influencing the homeostasis of inflammatory cells, including macrophages, neutrophils, and lymphocytes, plays critical roles in the pathogenesis of inflammation [14]. These data support a notion that by modulation of autophagy response the severity and activity of immune cells and pro-inflammatory cytokines could be controlled appropriately.

Studies have been documented that *Atg16L1* or *Atg7* deficient macrophages increased the production of IL-1β and IL-18 in response to inflammatory stimulation by provoking TLR 3/4 signaling pathway. As well, the TLR signaling can also promote autophagosome maturation and autophagolysosome formation through the activity of factors ATG5 and ATG7 that enhance sequestration and abscission of the ingested organism in macrophages/monocytes lineage [15].

### Regulation of pro-inflammatory cytokines by autophagy

Autophagy has the potential to regulate the secretion of cytokines from immune cells [16]. The regulation of the IL-1 family, especially IL-1B cytokine, is required for understanding inflammation status. Probably the well-documented aspect of the interaction between autophagy and inflammation is represented by the role of autophagy on induction of inflammasome and IL-1B secretion. Even, autophagy regulates IL-1β secretion. In this regards, Saitoh et al. have been reported that knockout Atg16L1 in mice macrophages increased the production of IL-1B after stimulation with bacterial lipopolysaccharide [17]. Monitoring the role of autophagy in human cells proved that the inhibition of autophagy led to increased production of IL-1B, indicating an important role of autophagy in the dynamic and biogenesis of IL-1B. Recently, it has been documented that autophagy inhibition through the suppression of Atg7 or Beclin-1 or treatment with the 3-Methyladenine (an autophagy inhibitor), in macrophages or dendritic cells, stimulates the secretion of IL-1β [18]. Similarly, autophagy also was found to regulate the secretion of cytokines such as IL-6, IL-18, and TNF-α. Autophagy inhibition stimulates IL-18 production coincided with a reduction of IL-6, − 8 and TNF-α production [19]. In the case of autophagy promotion, the activity of NF-ƙB is inhibited by selective degradation of BCL10 complexes [20]. Many mechanisms have been suggested to mediate these anti-inflammatory properties of autophagy. Defective autophagy leads to an accumulation of depolarized mitochondria, that release inflammasome activators such as mtDNA or ROS [21]. Additionally, autophagy may also eradicate aggregated inflammasome structures that lead to diminishing pro-inflammatory responses [22]. As a result, these data proposed that autophagy and inflammation are interlaced processes and any disorganizations in the multiple crosstalks between these two processes can have critical consequences for the pathogenesis and treatment of AS and other inflammatory conditions.

### Role of autophagy in atherosclerotic cells

Three different cells type are important for the initiation and development of AS: macrophages, SMCs and ECs. All of these cells could express autophagic markers [23].

#### Macrophages

**Fig. 2:**
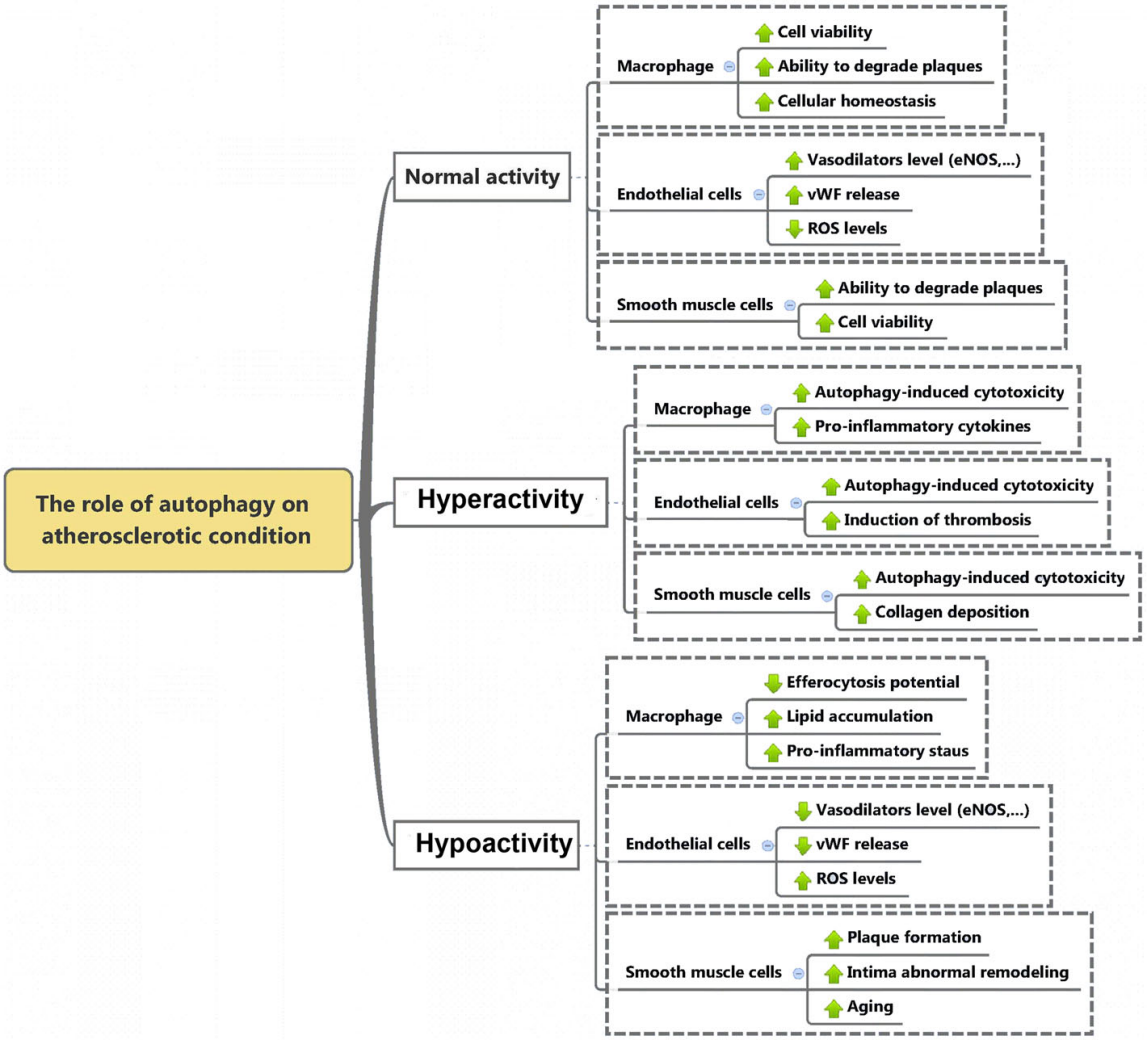
Effect of autophagy status on different cell types involved in AS. Normal autophagy flux in vascular system involved in cellular hemostasis but abnormal autophagy activity, either hyper- or hypo-activation, results in cellular imbalance and finally, cell death and AS plaque instability

Are known to play a pivotal role in AS and involved in the clearance of cholesterol deposits in vascular tissue at early stages. This section mainly discusses the close relationship between autophagic status in macrophage against AS. After the onset of atherosclerotic changes, circulating monocytes move into sub-endothelium of vessel walls and convert into macrophages, which subsequently turn into foam cells filled by oxLDL [2]. Foam cells are indicative of atherosclerotic lesions. Macrophage autophagy is known to play an important protective role in AS [24]. In line with this statement, the inhibition of autophagy in macrophages activates plaque destabilization and thereby necrosis is initiated through the luminal surface. In this regards, the induction of autophagy in macrophages by mTORC1 inhibition results in stabilization of atherosclerotic plaque [25]. It seems that the activation of C1q/CTRP9, a pro-inflammatory agent, during atherosclerotic changes could trigger the autophagy-related signaling pathway in foamy macrophages and pro-inhibits subsequent atheroma formation in *Apolipoprotein E* deficient mice [26, 27]. Sergin et al. found the positive effects of trehalose administration on autophagy and AS by induction of a lysosomal biogenesis factor TFEB in mice macrophage cells in vivo. These data support the athero-protective role of autophagic activity in macrophages (Fig. 2).

#### SMCs

Are the major component of the vascular system and acquire phenotypic shift during AS progression such as synthetic, macrophage-like or osteochondrogenic phenotypes [28]. SMCs are considered to play a crucial role in atherogenesis processes. Autophagy, by regulating vascular SMC phenotype, exerts a critical role in AS [29]. Normal autophagic activity in SMCs is related to cell survival and plaque stability but excessive autophagic activity results in SMC death and plaque destabilization [5, 30]. It has been demonstrated that exposure of SMCs to modest concentrations of oxLDL (10–40 μg/ml), occur in early stages of AS, increase autophagy that acts as protective mechanism, while exposure to high concentrations (≥60 μg/ml), happen in later stages of AS, leading to a decrease protective role of autophagy and enhancement of autophagic-induced cell death, authorizing that the stress response stimulated by autophagy becomes malfunctioned when a certain borderline of cell injury is achieved [31]. Recently, it has been documented that inhibition of autophagy by knockout of Atg7 in SMCs of the animal model show detrimental effects such as higher senescence, neo-intima formation (a new or thickened layer of arterial intima formed especially in AS by migration and proliferation of cells from the media) and atherogenesis [32]. In addition to the role of defective autophagy on plaque formation, plaque instability and rupture were also indicated in *Atg7* and *Apolipoprotein E* deficient mice. Histological examination revealed an increase in the number of SMCs with an aborted autophagy and cholesterol crystals and arthomas in tunica media of carotid artery [33]. Feeding of *Atg7* and *Apolipoprotein E* deficient mice with Western diet increased the number of autophagosomes inside the SMCs, indicating an aborted autophagy response [34].

Autophagy and mitophagy in SMCs can be induced by oxLDL, which is associated with accumulation of PINK1 and Parkin at the outer membrane of the damaged mitochondria, and promote cell survival and inhibit against VSMC apoptosis similarly to macrophages [35]. In addition, 4-hydroxynonenal, a lipid peroxidation product, can activate autophagy in VSMCs [36]. SMCs autophagy also may regulate by different cytokines such as TNF-α and osteopontin and growth factors such as the PDGF [37]. PDGF secreted by several cell types during vascular injury protects against cellular death via activation of autophagy (Fig. 2) [29].

#### ECs

Are one of the most dynamic cells in the body. Functional changes in these cells are associated with cardiovascular diseases. There is a growing body of literature attempting to understand the effect of autophagy on vascular pathophysiology. It is well known that autophagy plays an important role in accurate ECs function [28]. Studies have been demonstrated that autophagy in ECs induces endothelial eNOS expression and subsequently, NO accessibility decrease oxidative stress and inhibit inflammatory cytokine production [38, 39]. Also, it is well established that the detrimental effects of angiotensin-II on endothelial dysfunction may be limit by the autophagic system [40]. Evidence showed that the modulation of specific miRNAs, namely miRNA876, in collaboration with an apoptotic agent could exacerbate the detrimental effects of AS on the luminal surface [41]. In addition, autophagy in ECs regulates the secretion of endothelial von Willebrand factor that plays a role in the coagulation process [42]. The present of autophagic activity in ECs of atherosclerotic plaques also demonstrated recently [43]. Also, it has been observed that oxidative stress, which occurs in the atherosclerotic environment initiate autophagy in human ECs [44]. Similar to macrophages and SMCs, the excessive autophagic activity can mediate autophagic-induced cell death in ECs that provoking instability of plaque [34]. Recently, Vion and colleagues demonstrate that inefficient autophagy contributes to the development of atherosclerotic plaques promotes inflammation, apoptosis, and senescent phenotype in ECs [45]. Because of the critical role of endothelial malfunction in the pathogenesis of vascular-related disease such as AS, it would be told that the distraction of the autophagy mechanism in ECs has noteworthy contributions. In a few words, documents are available that the stimulation of autophagy activity safeguards versus cellular damage in all cell types engaged in atherosclerotic disease (Fig. 2) [11].

### Protective effects of autophagy in AS

The role of autophagy in AS is not well understood yet. Autophagy is recognized in vascular SMCs in the fibrous cap of the late lesion as one of the important mechanisms for stabilizing the lesion. Autophagy maintains plaque cells against oxidative stress, a hallmark of advanced atherosclerotic lesions, by degrading the multifunctional materials, especially polarized mitochondria prior to cytochrome C release namely mitophagy [11]. Thereby, favorable autophagy of the damaged components prohibits the development of apoptosis and contributes to cellular retrieval. However, acute or chronic oxidative stress results in an intracellular increase of ROS that damage the lipid membrane of lysosomes. Lysosomes with impaired membrane are not able to fuse with autophagosomes carrying damaged components and thereby the release of potent hydrolases and some degree of cellular damage happen. As a conclusion, If autophagy is not involved adequately as part of the oxidative stress response in atherosclerotic plaques, or when oxidative damage overwhelms the cellular defenses, probably, the cell will die via the activation of apoptosis [11]. Another example of autophagy protection in AS is shown in a recent pharmacological study by using 7-ketocholesterol and statins. Statins protect patients from ischemic heart disease such as MI, although they are known to induce SMCs apoptosis in a dose-dependent manner. Other modulatory effects are related to statins. In the distinct condition, the biosynthesis of geranylgeranyl increases the cellular distribution of cellular geranylgeranyl diphosphate which inhibits autophagic response by the restriction of GTPase Rab proteins activity. It was shown that statins possess the potential to inhibit hydroxymethylglutaryl-CoA reductase bioactivity which decreases the amount of protein farnesylation or geranylation rate sequestration and abscission of material outside the cells [46]. The death of SMCs induced by low levels of statins attenuated by the autophagy inducer 7-ketcholesterol. In this way, Martinet W and colleagues demonstrated that fluvastatin fails to raise Caspases activity in SMCs that treated with 7-ketocholesterol. This study supports the notion that the initiation of autophagy interferes with the statin-induced apoptotic pathway [47]. Although the details of this suppression have not been fully elucidated yet, it is believed that sequestration of damaged mitochondria in the autophagosomes prevents apoptosis by incarceration of apoptosis-inducing protein and pro-apoptotic factors such as cytochrome C [48]. In recent years, the inevitable role of autophagy has been documented as a major role in late-stage quality control of Apo-lipoprotein B protein secreted by the liver. Accumulation of lipoproteins containing apoB in the arterial vessel walls is an important starting event in the progression of AS [49]. Other effectors could directly participate in the activation of autophagy signaling pathway during AS. For example, the positive role of Statins family member such as simvastatin was documented in coronary artery myocytes under the risk of atheroma formation by ox-LDL [50]. In this regard, simultaneous administration of Simvastatin with ox-LDL in J774A.1 murine macrophage cell line could provoke the mTOR signaling pathway, prohibit lipid accumulation by delivering to the lysosomes [51]. It seems that the protective effects of autophagy diminish by aging coincided with alteration in the level of autophagy effectors. For instance, elevated levels of P62 with reduced Belin-1 in old mice were determined to show the suppression of autophagy and autophagolysosome formation [38].

### Deleterious effects of autophagy in AS

Despite its protective role, autophagy is responsible for the formation of ceroids in AS that is yellow-to brown insoluble protein complexes associated with oxidized lipids found in all atherosclerotic lesions. Hydrogen peroxide produced by mitochondria and other organelles can permeate into the lipid membrane and enter the lysosomes. Due to the existence of iron ions in lysosomes, cells entering autophagy degradation, the interaction between iron ions and hydrogen peroxide is started via Fenton reactions, resulting in the production of hydroxyl radicals that induce lipid peroxidation and finally intermolecular crosslinking and ceroid formation [52]. It has been reported that iron and ceroid deposits co-localize in foam cell-like macrophages or SMCs of advanced atherosclerotic plaques. Many cells in late human plaques contain a large number of ceroid containing lysosomes and a variety of lysosomal enzymes that degrade ceroid. These lysosomal enzymes are lost for useful purposes (eg, for the degradation of newly autophagocytosed material), resulting in impaired autophagy and the induction of apoptosis [52]. Impaired autophagy stimulates the accumulation of dysfunctional mitochondria, promoting ROS content and increased ceroid formation. Continuous degradation of iron-containing materials through autophagy results in the production of hydrogen peroxide and the peroxidation of the lysosomal membrane, consequently rupture of their membrane, especially under severe oxidative stress conditions release destructive lysosomal enzymes without any control [52]. In conclusion, the released lysosomal enzymes attack normal proteins and mitochondria. As a matter of fact, cytochrome C is released and per se amplifies the apoptotic program [53]. In contrast to the basal level of autophagy, excessive activated autophagy may induce apoptotic death of SMCs that induce the formation of the fibrous membrane and while reduces normal collagen synthesis, thereby deepening plaque instability. In addition, the apoptotic death of ECs is seen by the over-activity of autophagy, having a detrimental effect for the structure of the plaque and formation of regional thrombosis seen in clinical events [54, 55]. Also, In AS, excessively stimulated autophagy can result in EC death that can contribute to plaque destabilization and maintaining the inflammatory status of the plaque [34]. Despite the normal activation of autophagy on reversal age-related pathologies [56], the overexpression of autophagy initiator such as beclin-1 intensifies the pathological changes and contributes to autophagic cell death under the situation of continuous injuries [57].

Autosis has recently defined a new form of autophagy-dependent cell death that indicated by increased cell-substrate adhesion, focal swelling of the perinuclear region, and disassociation of endoplasmic reticulum with an impaired membrane Na+/K + -ATPase activity [58]. It seems that the autosis could be triggered by autophagy-inducing conditions such as peptides, starvation, and hypoxia-ischemia. Results of the newly-published document confirmed that cardiomyocytes autosis occurs in the heart during I/R and contributes to overall myocardial injury in response to I/R [59, 60]. Concurrently, Nah et al. recently demonstrated that autosis may be activated by the accumulation of autophagosomes, due to the imbalance between TFEB and lysosome function during the late phase of I/R [61].

### Modulation of autophagy in the treatment of AS by phyto-compounds

Hitherto, we described autophagy and its role in all cell types involved in AS. Many fundamental investigations and clinical trials have been managed to address autophagic machine to treat AS. In reference to these studies, numerous autophagy stimulators have been shown to be effective for the mitigation of AS severity Table 1. With this in mind, we propose that stimulation of autophagy can be as a promising treatment strategy for AS and targeted therapy of autophagy may be operative and hopeful therapies for AS treatment.

**Table 1:** Anti-atherosclerotic effects of different compounds by modulation of autophagy

*Compounds*	*Functions*	anti-atherosclerotic effects	*References*
Everolimus	An mTOR inhibitor, autophagy inducer,	depletion of plaque macrophages, improvement of cholesterol efflux, lowering systemic and local inflammation, inhibiting intra-plaque neovascularization, enhance plaque stability, reduce intimal thickening	[57–60]
Resveratrol	An mTOR inhibitor, autophagy inducer, anti-oxidative and anti-inflammatory element,	facilitated the efferocytosis of apoptotic cells, decrease of atherosclerotic size and density of plaque, reducing layer thickness, inhibition of age related changes	[61–64]
Berberine	activation of the AMPK/mTOR, autophagy inducer, antioxidant, anti-inflammatory, anti-hyperlipidemic, anti-microbial	inhibit inflammatory reactions,	[65, 66]
Β-arrestins	autophagy inducer,	reduce neointimal hyperplasia	[67, 68]
CB2R agonists	autophagy inducer, inhibits NLRP3 inflammasome,	Inhibition of inflammatory response	[70, 71]
statins	mTOR inhibitor, autophagy inducer, anti-inflammatory properties	improvement of EC function, plaque stabilization	[72]
Metformin	stimulation of AMPK, autophagy inducer, anti-inflammatory function,	decrease vascular problems, suppresses vascular senility	[73, 74]

It has been well-documented that mTOR inhibitors have therapeutic significance in many types of disease. Everolimus, one of the rapamycin derivatives (rapalogs) and the mTOR inhibitors agents, is the most studied and famous autophagy inducers. These agents elicit a cluster of anti-atherosclerotic effects, including depletion of plaque macrophages, improvement of cholesterol efflux, lowering systemic and local inflammation, and inhibiting intra-plaque neovascularization [62]. Everolimus heightened the clearance of toxic materials by inhibiting mTOR and then, inducing autophagy [63]. Because of this outstanding potency of Everolimus, its role in treatment for AS has been extensively studied and reported. Based on this evidences, everolimus enhance plaque stability, reduce intimal thickening [64, 65]. These promising data are slightly eclipsed by their adverse effects on blood lipid and glucose levels, so that combined therapy with statins or metformin is desirable.

#### Resveratrol

Another mTOR inhibitor, and autophagy inducer is a plant-derived polyphenol, anti-oxidative and anti-inflammatory elements and plays a protective role against various types of diseases AS. Resveratrol, by inducing autophagy, have excellent anti-AS properties [66]. Recently, an in vitro study confirmed that resveratrol facilitated the efferocytosis of apoptotic cells by activating autophagy [67]. In addition, resveratrol supplementation in rabbit model results in a decrease of atherosclerotic size and density of plaque and reduction in layer thickness [68]. The age-related changes in arteries of rhesus monkeys due to autophagy insufficiency was also shown to be removed after resveratrol administration [69]. This excellent property of resveratrol proposing that resveratrol may serve as a novel therapy for the treatment AS disease.

Numerous studies reported that Berberine, extracted from Coptis, has been demonstrated to be a potential therapy for the treatment of several types of diseases owing to its different pharmacological properties including antioxidant, anti-inflammatory, anti-hyperlipidemic, anti-microbial and other outstanding properties [70]. Fan and colleagues, recently, reported the mechanism of protective effect of berberine against AS. They demonstrated that berberine induces autophagy process, mediating through the activation of the AMPK/mTOR signaling pathway, and consequently, inhibit inflammatory reactions [71]. These data provide new vision into molecular mechanisms of berberine’s and its relationship with autophagy as a therapeutic agent in AS treatment.

According to previously published data, there is a close relationship between anti-oxidant property and autophagy modulation in cells after exposure to berberine and resveratrol. Both phyto-compounds have the potency to exert their effects by engaging different mechanisms on target effectors. For example, resveratrol induces AMPK, SIRT1, LC3-II/I ratio and activates Akt-BCl-2 and suppress the mTOR and P70S6K while berberine suppresses mTOR, ERK1/2 MAPK, Beclin, and SIRT1. These effects stand for a fact that the modulation of autophagy could be initiated by different effectors and signaling pathway. Although, these compounds activate the autophagic responses in target cells the genomic and proteomic profile of distinct cell types differ after treatment with both agents [72]. The activation of MAPK by both resveratrol and berberine could increase fatty acid oxidation and insulin sensitivity thereby these modalities contribute to the production of ATP and neutralization of reactive oxygen species. The induction of autophagic response and scavenging free radicals seem to be critical points to reduce the pathologies of atherosclerotic changes.

#### Β-arrestins

Are a versatile, multifunctional and small family of adapter proteins that are best known for their potential to desensitize and deactivated GPCRs, but also regulate a numerous of cellular fundamental function [73]. It has been established that β-arrestin-1 activated Beclin1-dependent autophagy in neurons, revealing a neuroprotective role for β-arrestin-1 in cerebral ischemia [74]. Also, previously, it has been demonstrated that silencing of β-arrestin-1 significantly promotes the severity and progression of AS by reducing neointimal hyperplasia in SMCs. These data suggested that β-arrestin-1 played a protective role in mitigating AS via activating autophagic response [75].

Another promising autophagy inducer agents are cannabinoid receptor 2 agonists that induce the autophagic activity, inhibits NLRP3 inflammasome activation and initiation in microglia and contributing to the alleviation of experimental autoimmune encephalomyelitis (EAE) [76]. In combination with the previously documented protective effects of CB2R in AS [77]. CB2R agonists are another cluster of autophagy-induced elements that are probably to be valuable for the AS treatment.

The statins, celebrated for their pleiotropic effects such as anti-inflammatory properties, improvement of EC function, and plaque stabilization, have been reported to stimulate autophagy in SMCs through inhibition of the mTOR pathway [78].

Metformin, an anti-diabetic drug, activate autophagy and inhibits mTOR via stimulation of AMPK. Metformin has been validated to decrease vascular problems in diabetic patients [79]. Metformin suppresses vascular senility and has been demonstrated to mitigate AS in diabetics [80]. Metformin also promotes mitochondrial quality by decreasing mitochondrial disintegration in by AMPK-dependent manner [81]. recently, it has been reported that the anti-inflammatory function of metformin on ECs is autophagy-dependent [82] but more studies must be developed to determine whether the profitable cardiovascular properties of metformin are linked to autophagy activation. Therefore, pharmacologic Interventions directed toward recovering normal autophagic flux may have targeted therapeutic potential in related diseases.

## Conclusion and future perspectives

Considering the double-edged sword activity of autophagy, it seems that precise detection of autophagy role in the progression and/or inhibition of AS must be considered in the favor of therapy. The possible modulation of autophagy effectors by the different pharmacological agent is needed to investigate in future investigations. Elucidating the pattern and trend of autophagic response in different cell types could help us to predict the cell resistance/sensitivity to ongoing pathological changes. Since the impact of several forms of autophagy, macro/micro/chaperone-mediated), and aggrephagy, mitophagy, lipophagy, and xenophagy were not completely addressed in AS, some further investigations are needed to unveil modulatory effects during the progression and suppression of AS. It seems that the selection of autophagy modulation must be considered according to the development, progression, and pathology of AS. Any approaches targeting the autophagy modulation in the advanced atherosclerotic changes not only does not amoleriate the atherosclerotic changes but also could exacerbate the pathological changes. Autophagy could reverse the atherosclerotic changes in the early stages while the dictation of autophagic responses in developing pathologies could intensify the AS outcomes. Therefore, the oriented regulation of autophagy is helpful to circumvent these limitations.

## Abbreviations

AS: Atherosclerosis
Atg: Autophagy-associated genes
CMA: Chaperone-mediated autophagy
CVD: Cardiovascular disease
ECs: Endothelial Cells
eNOS: Nitric oxide synthase
GPCRs: G protein-coupled receptors
HSC70: Heat shock cognate 71 kDa protein
I/R: Ischemia/reperfusion
IL-1: Interleukin-1
LAMP2A: Lysosomal-associated membrane protein type 2A
MI: Myocardial infarction
mtDNA: Mitochondrial DNA
mTOR: Mammalian target of rapamycin
NF-ƙB: Nuclear Factor of Kappa light polypeptide gene enhancer in B-cells
NO: Nitric oxide
oxLDL: Oxygenized low-density lipoproteins
PDGF: Platelet-derived growth factor
ROS: Reactive oxygen species
SMC: Smooth muscle cells
TLR: Toll-like receptor
